# Why Does Mental Health Not Get the Attention It Deserves? An Application of the Shiffman and Smith Framework

**DOI:** 10.1371/journal.pmed.1001178

**Published:** 2012-02-28

**Authors:** Mark Tomlinson, Crick Lund

**Affiliations:** 1 Centre for Public Mental Health, Department of Psychology, Stellenbosch University, Stellenbosch, South Africa; 2 Centre for Public Mental Health, Department of Psychiatry and Mental Health, University of Cape Town, Cape Town, South Africa

## Abstract

Mark Tomlinson and Crick Lund analyze why mental health does not garner the international attention, political priority, or funding that it deserves, and offer suggestions to improve the visibility of global mental health.

**Summary Points**

- Despite a high disease burden, mental illness has thus far not achieved commensurate visibility, policy attention, or funding.
- We apply the framework of Shiffman and Smith in order to understand the current position of global mental health with regard to generating funding and political priority.
- While significant progress has been made in terms of prioritising mental health globally, debates around the definition of mental illness, and the continued impact of stigma, remain.
- We make a number of recommendations to increase the visibility and policy priority of mental health as a global issue.

## Introduction

The lifetime prevalence of mental disorders has been estimated to be between 12.2% and 48.6% globally [1]. More than 13% of the global burden of disease for mental disorders is due to neuropsychiatric disorders, and over 70% of this burden lies in low- and middle-income countries [2]. Suicide is one of the leading causes of death globally for all ages [3]. Despite this burden, mental illness has thus far not achieved commensurate visibility, policy attention, or funding, particularly in low- and middle-income countries [4].

Shiffman and Smith [5] have developed a framework of analysis that attempts to understand why some global health initiatives are more successful in generating funding and political priority than others. The framework has been applied most prominently to maternal mortality and newborn survival [5],[6]. Global mental health is one initiative that is attempting to garner an increased share of international funding as well as prioritisation by political leaders. In this essay, we will use the Shiffman and Smith framework to demonstrate that while some significant strides have been made, mental health still faces major challenges in establishing itself as a global initiative with meaningful political priority. We will conclude with a discussion of the way forward for the global mental health movement, and make some suggestions about how this aim can be furthered.

## Global Mental Health and the Shiffman and Smith Framework

Shiffman and Smith [5] have argued that a health issue gains political priority when three conditions are met: (1) country political leaders as well as international leaders publicly (as well as privately) express support for the issue, and do so in a sustained fashion; (2) policies are enacted to address the problem; and (3) resources (appropriate to the disease burden) are allocated to the issue. In the case of mental health, none of these conditions is currently being met in a substantial way. There is little public (or private) support for mental illness as a global priority. At the recent United Nations General Assembly Special Session on Non-Communicable Diseases, it was only through sustained lobbying from the World Health Organization, the World Federation for Mental Health, and others that mental health was even mentioned, but not as one of the four priority conditions. With regard to the issue of policies enacted, as many as 44% of African countries do not even have a mental health policy, and 33% do not have a mental health plan [4]. In relation to resources, recently published data from the World Health Organization's “Mental Health Atlas 2011” indicate that little has changed in the allocation of resources for mental health care during the last ten years, particularly in low- and middle-income countries [4]. As a global median, 2.8% of health budgets are allocated to mental health, with wide variation (from 0.53% of low-income countries' to 5.10% of high-income countries' budgets), indicating that, proportionally, lower income countries spend a smaller percentage of their health budget on mental health [4]. There is a robust correlation (*r* = 0.78) between gross national income per capita and mental health expenditures per capita [4]. Yet even in rich countries, when health budgets are cut, quite often the first area to be cut is mental health. In the United States, US$2,100,000,000 has been cut from mental health budgets over the last three years, and further cuts are expected for 2012.

The Shiffman and Smith framework consists of four components: actor power, the ideas used to describe the issue, the context within which the actors are operating, and the characteristics of the issue itself [5],[6].

### Actor Power

Actor power in this framework consists of three components: cohesive leadership (Shiffman and Smith found that having a group of no more than 15 persons leading the initiative was a significant factor contributing to the rise of global attention to newborn survival—the extent to which this will be true for other health concerns remains to be seen); a guiding institution (either an organization or a more informal network, but one connected by similar values and goals); and the mobilization of civil society in order to advocate at national and international levels [5]. In the case of global mental health, over the last decade, a core group of individuals and their associated institutions have driven the publication of the “World Health Report 2001—Mental Health: New Understanding, New Hope”, which focused on mental health for the first time [7], “The WHO Mental Health Policy and Service Guidance Package” (2003–2005) [8], the World Health Organization Mental Health Gap Action Programme (mhGAP) (2008), and related initiatives such as *The Lancet*'s Series on Global Mental Health (2007 and 2011) [9],[10], the *PLoS Medicine* series Packages of Care for Mental Health Disorders in Low- and Middle-Income Countries [11], and the *Nature* article “Grand Challenges in Global Mental Health” [12]. Taken together, these milestones have shown a relatively cohesive body of academic leadership in this field. However, despite the launch of related advocacy initiatives such as the Movement for Global Mental Health and the World Federation for Mental Health's “Great Push for Mental Health”, there has not been sufficient mobilization of civil society to advocate with adequate power at national and international levels, as was evident in the outcome of the UN non-communicable diseases summit. While there are numerous user groups and organisations that advocate for greater public priority for mental disorders, it is only in some countries such as Australia (National Mental Health Consumer & Carer Forum; http://www.nmhccf.org.au/) where they have substantive power.

### Ideas

Ideas in this framework refer to how the issue is characterised and described in trying to draw attention to it. Shiffman and Smith argue that some health campaigns are easier to promote than others because the diseases they address are seen to be more harmful (for example, neonatal mortality, with 4 million global deaths per year) and have more cost-effective and simple evidence-based solutions [5]. In the case of global mental health, it has been difficult to develop a common construct that can be promoted. There have been some gains in this area, for example, through the landmark publications listed above, but dissenting and critical voices remain. Many continue to question what they consider to be the universalistic nosological assumptions of current diagnostic instruments [13]. There are two distinct diagnostic classification systems (Diagnostic and Statistical Manual of Mental Disorders and the International Classification of Diseases), and more recently other approaches have emerged, “transdiagnostic” or “modular” approaches that focus on the similar underlying pathological processes that cut across diagnostic categories [14]. The mental health care community currently lacks a widely accepted framework on the classification, causes, and treatment of mental ill health.

More broadly a distinction between “internal” and “external” debates has yet to emerge in global mental health. “Internal” debates might include rigorous interrogation of the complex issues underlying the diagnosis of mental illness and the nosological systems that need to be developed to facilitate accurate, culturally valid diagnoses. Currently, these debates are being presented in the “external” arena of global policy debate, contributing to policy and political leaders' confusion as to what the priorities for mental health should be, and how to define, measure, and narrow the treatment gap. These debates should ideally occur “internally”, with a more unified position about how to advocate for mental illness when presenting to policy makers, politicians, or donors (the external frame).

### Context

Context in the Shiffman and Smith framework is the environment in which the actors operate and includes the ability of the global actors to take advantage of policy windows to influence decision makers. The United Nations high level meeting on non-communicable diseases was just such a policy window, but global mental health actors were not able to take full advantage of this opportunity. This may come to be seen as an important missed opportunity. There may be many reasons for this, such as the unwillingness of key leaders in the non-communicable diseases summit initiative to give space to mental health, the lack of a groundswell of community-based advocacy initiatives for mental health, and perceptions that the burden of mental illness and attendant interventions are difficult to quantify [15]. For newborn survival, in contrast, an informal network of no more than 15 prominent researchers were able to act as one and were able to take advantage of Millennium Development Goal 4 as a policy window to effectively put newborn survival firmly on the global health agenda [6]. Mental health is completely ignored in the Millennium Development Goals (as are most non-communicable disorders), despite compelling evidence that mental health is implicit in many of these targets [16].

### Characteristics of the Issue

The characteristics of the issue being addressed include the extent to which there are credible indicators that can be used to assess severity and to monitor progress and the size of the burden, as well as an evidence base on cost-effective interventions that can be implemented at scale. In the case of mental health, there is an increasing body of evidence of credible indicators and of the disease burden of mental illness globally [9]. There is also reasonably robust evidence on cost-effective interventions that can be delivered in low- and middle-income countries [17]. However, despite evidence on which interventions work, the evidence on how these interventions can be delivered in routine low-resource settings remains sparse, although a recent initiative—the Programme for Improving Mental Health Care (PRIME)—aims to provide crucial data in this regard.

## The Way Forward

Significant strides have been made in recent years towards ensuring a greater prominence for mental health on the global health stage. *The Lancet*'s Global Mental Health series [10], the *PLoS Medicine* Packages of Care series [11], and the recent Grand Challenges in Global Mental Health article [12] are all important initiatives that have raised the profile of mental health. While significant funding for mental health has not been forthcoming from global health foundations such as the Bill & Melinda Gates Foundation, recent initiatives from Grand Challenges Canada, the UK Department for International Development, and the US National Institute of Mental Health have begun to redress this imbalance. It is possible that the Grand Challenges in Global Mental Health initiative may serve as a rallying point that might facilitate some cohesion amongst the policy community. Currently the World Health Organization, the World Federation of Mental Health, and journals such as *PLoS Medicine* and *The Lancet* are at the forefront of attempts to increase awareness of mental illness. Other initiatives include the New York University Learning Network for Global Mental Health, which seeks to build capacity for systems that enable scaling up of mental health care [18]. The Centre for Global Mental Health was established in 2009 and has the potential to serve as a unifying global mental health research and advocacy network. There is also some emerging epidemiological evidence of population-level impacts of service delivery, for example, in Australia some progress has been made with lowering suicide rates [19], and in the US temporal associations have been demonstrated between fluoxetine prescriptions and declining suicide rates [20], although suicide rates are widely acknowledged to have multiple social and economic determinants.

On the other hand, there has been little change in the perception of the intractability of mental illness [15], combined with the related problem of stigma associated with mental illness [21]. Significant efforts are underway to address stigma, notably within the INDIGO Network, which spans 27 countries and is investigating stigmatization of and discrimination against the mentally ill [22]. Finally, while newborn survival advocates made good use of the policy window afforded by the Millennium Development Goals, global mental health has had difficulty making the case for its importance across numerous Millennium Development Goals [16]. In the light of this discussion, we would like to suggest a few steps (see also Box 1).

**Box 1. Recommendations for Increasing Attention to Global Mental Health**

1. Greater community cohesion and international governance structures need to be developed to contribute to a more unified voice regarding global mental health.
2. A common framework of integrated innovation is needed to ensure that global mental health speaks in the language of national and international policy makers.
3. For global mental health to gain significant attention, a coherent evidence base for scalable interventions that can be shown to have an impact at the structural level—on economic development and human well-being—is central.
4. A social justice and human rights approach is important.
5. Current innovative strategies for addressing stigma need to be evaluated and expanded.

First, greater community cohesion and international governance structures need to be developed to contribute to a more unified voice regarding global mental health. International organisations such as the World Health Organization, the World Federation for Mental Health, and the Movement for Global Mental Health, as well as national organisations, need to become a united force, for example, through a unified organisational network that delivers clear, consistent, and well-timed messages for policy and public consumption. Involvement of mental health care users, their families, and civil society is crucial in this regard [23]. Unless this is done, it is likely the next “policy window” will be missed.

Second, we need to develop an effective frame of integrated innovation that will ensure that global mental health speaks with a united voice, and does so in the language of national and international leaders, in order to ensure public and private support for the issue. This includes engaging in frank and open discussion with dissenting voices in order to build a coherent and common language.

Third, it is possible that for mental health to gain significant attention, it is not enough to convince people that it has a high disease burden, and that there are deliverable and cost-effective interventions. Shiffman [24] argues that HIV/AIDS was successful in gaining significant issue attention because it was able to convince national and international political leaders that HIV/AIDS was a threat to human well-being and national security, and that getting HIV/AIDS under control was central to national economic development [24]. Global mental health must similarly demonstrate its social and economic impact. A coherent evidence base for scalable interventions that can be shown to have an impact at the structural level—on economic development and human well-being—is central [25]. This is the language of most policy makers.

Fourth, a social justice and human rights framework is also crucial for this cause. Current initiatives such as the World Health Organization's QualityRights Project [26] and the “WHO Resource Book on Mental Health, Human Rights and Legislation” [27], as well as a forthcoming volume on mental health and human rights [28], are important steps in this respect.

Fifth, stigma continues to contribute to the notion that mental illness is an intractable (or in some circles negligible) public health problem [21]. This issue needs concerted attention, and innovative approaches need to be developed to address stigma in a systematic and evidence-based manner.
